# Supplementary material for: Quality of Clinical Guidelines on Oral Care for Children with Special Healthcare Needs: A Systematic Review
Source: Int J Environ Res Public Health. 2023 Jan 17;20(3):1686. doi: 10.3390/ijerph20031686 (PMC9914149; doi:10.3390/ijerph20031686)
Supplement: Supplementary file 1 [file ijerph-20-01686-s001.zip › ijerph-1991957-supplementary.pdf]

Section S1: Guideline's appraisal- electronic search strategies

MEDLINE (OVID) search strategy

1. Disabilities.mp.
2. special healthcare need.mp.
3. special health need.mp.
4. exp Disabled Children/
5. exp Intellectual Disability/
6. exp Developmental Disabilities/
7. medical compromised.mp.
8. medical ill.mp.
9. limitation.mp.
10. or/1-9
11. exp Child/
12. children.mp.
13. exp Child, Preschool/
14. exp Adolescent/
15. exp Young Adult/
16. or/11-15
17. 10 and 16
18. (oral adj6 care$).ti,ab.
19. exp Dental Care/
20. Management.mp.
21. Treatment.mp.
22. ((oral or mouth) adj5 care).ti,ab.
23. or/18-22
24. 17 and 23
25. exp Consensus Development Conference/
26. exp Guideline/
27. exp Guidelines as Topic/
28. exp Practice Guideline/
29. Practice Guidelines as Topic/
30. Health Planning Guidelines/
31. (standards or guideline or guidelines or guidance$).ti,kf,kw.
32. ((practice or treatment$ or clinical) adj guideline$).ab.
33. or/25-32
34. 24 and 33

Section S2.

Additionally, the following websites were searched thoroughly to find clinical guidelines for children with SHCNs in dentistry:

1. The National Institute for Health and Care Excellence (NICE) (https://www.nice.org.uk/)
2. The Royal College of Surgeons of England (https://www.rcseng.ac.uk/)
3. The British Society of Paediatric Dentistry (http://bspd.co.uk/)
4. The Scottish Dental Clinical Effectiveness Programme (SDCEP) (https://www.sdcep.org.uk)
5. British Society for Disability and Oral Health (http://www.bsdh.org/)
6. American Academy of Paediatric Dentistry (http://www.aapd.org/)
7. Special Care Dentistry Association (http://www.scdaonline.org/)
8. US National Guideline Clearinghouse (https://www.guideline.gov/)
